# Long-term health outcomes of preterm birth: a narrative review

**DOI:** 10.3389/fped.2025.1565897

**Published:** 2025-04-23

**Authors:** Faith Gette, Sumera Aziz Ali, Matthew S. P. Ho, Lindsay L. Richter, Edmond S. Chan, Connie L. Yang, Emily Kieran, Cherry Mammen, Ashley Roberts, Kristopher T. Kang, Jonathan Wong, Shahrad R. Rassekh, Michael Castaldo, Kevin C. Harris, James Lee, Carol K. L. Lam, Natalie H. Chan, Sarka Lisonkova, Joseph Y. Ting

**Affiliations:** ^1^ Department of Pediatrics, University of Alberta, Edmonton, AB, Canada; ^2^ Department of Pediatrics, University of British Columbia, Vancouver, BC, Canada; ^3^ Department of Pediatrics, University of Toronto, Toronto, ON, Canada; ^4^ Department of Pediatrics, University of California at San Francisco, San Francisco, CA, United States; ^5^ Department of Obstetrics & Gynecology, University of British Columbia, Vancouver, BC, Canada

**Keywords:** preterm birth, neonate, perinatal epidemiology, health outcomes, narrative review

## Abstract

Despite a significant reduction in neonatal mortality due to advances in neonatal care, preterm birth (PTB) continues to pose a challenge due to the escalating incidence of long-term complications, which refer to health issues that persist or emerge beyond the immediate neonatal period. The impact of PTB, particularly in extremely preterm infants born before 28 weeks of gestational age, is not confined to the early years but extends across the lifespan, influencing physical, cognitive, and social development, as well as long-term health outcomes. These complications, which often persist from childhood into adulthood, span multiple systems and create a broad spectrum of health concerns. This comprehensive narrative review of literature delves into the breadth of well-characterized long-term complications associated with PTB, including neurodevelopmental, respiratory, cardiovascular, renal, gastrointestinal, and endocrine system disorders. By providing health care providers with a holistic understanding of the potential complications following PTB, this review aims to summarize the current literature and underscore the value of long-term monitoring strategies and proactive evaluations of this population. Our objective is to foster a clinical approach that anticipates these complications, enabling early interventions and better management of these at-risk infants.

## 1 Introduction

Preterm birth (PTB), defined as birth occurring prior to 37 weeks of gestation, encompasses various stages contingent on gestational age (GA): moderate to late preterm (32–37 weeks GA), very preterm (28 to less than 32 weeks GA), and extremely preterm (before 28 weeks GA) (1). In 2020, an estimated 13.4 million babies were born preterm, accounting for about 9.9% of all births worldwide (2). Notably, 15% of these preterm births occurred before 32 weeks of gestation, with 4.2% before 28 weeks and 10.4% between 28 and 32 weeks (2). The burden of PTB is disproportionately high in low- and middle-income countries (LMICs), particularly in Southern Asia and sub-Saharan Africa, which together contribute to 65% of global PTBs (2). Southern Asia, for instance, had the highest PTB rate globally (13.2%), with Bangladesh reaching a national rate of 16.2%, among the highest worldwide (2). Malawi also reported a high PTB rate of 14.5% (2). In contrast, PTB rates in high-income countries (HICs) are relatively lower, with Canada (8.0%) aligning closely with England (7.4%) and the USA (10.0%), while China reports a rate of 6.1% (2–5).

Similar to the differences in PTB rates between LMICs and HICs, there are notable disparities in survival outcomes (6). According to a global analysis by Cao et al., the age-standardized mortality rate of neonatal preterm birth declined in HICs between 1990 and 2019 (6), reflecting advancements in neonatal care, including improved respiratory support, surfactant therapy, and infection control measures (7). This declining trend in mortality rate was particularly evident in Bahrain, Greece, Japan, the UK, and the USA (6). However, in LMICs, particularly in Southern Sub-Saharan Africa, age-standardized mortality rate increased, indicating worsening neonatal survival despite global efforts to improve perinatal care (6). The progression of neonatal care, especially in HICs, has resulted in a global reduction in neonatal mortality associated with PTB by 47.7% from 1.27 million in 1990 to 0.66 million newborn deaths in 2019 (6). Consequently, a significant number of preterm infants can now survive into adulthood (8, 9). However, this decline has contributed to growing morbidity and long-term health risks for survivors (10, 11), as reinforced by population-based studies showing the lasting impact of PTB across the lifespan (12–16). For instance, a Norwegian cohort study following over 900,000 individuals found that those born extremely preterm had significantly higher risks of cerebral palsy, intellectual disability, and reliance on disability pensions in adulthood (14). Similarly, a German study of adolescents born before 27 weeks reported higher rates of psychological problems and lower health-related quality of life (13). A longitudinal cohort study from New Zealand also revealed that individuals born preterm were slower to reach their genetically determined height compared to those born full-term (16). The findings from these population-based studies highlight the persistent challenges faced by preterm survivors, emphasizing the need for long-term support and intervention.

Generally, the earlier the GA at birth, the more pronounced the medical, economic, and social consequences (14, 17). Hence, long-term consequences of PTB become a pressing public health concern. It is important to recognize that health outcomes extend beyond the immediate postnatal period, and that health is a continuum from intrauterine to postnatal conditions (11). Children and adolescents born prematurely also exhibit greater demand for specialized outpatient services, leading to higher long-term health care costs compared to their term-born peers (18). In the USA, the total estimated cost for PTBs in 2016 was approximately $25 billion and each PTB case incurred an average of $64,815 more in expenses compared to each term or post-term birth (19). In fact, during the first year of life, preterm children incur higher medical expenses compared to term infants due to the likelihood of various morbidities that require specialized care (20, 21).

While the initial hospitalization costs of preterm infants are well-documented (20, 21), emerging evidence highlights the sustained economic burden associated with PTB beyond infancy. A systematic review of economic consequences documented an inverse relationship between GA and healthcare costs, extending into childhood (22). Similarly, EPICure study showed that extremely preterm infants incurred higher public sector costs at age 11 compared to term-born peers (23). These findings are further supported by a European cohort study reporting an increased societal costs at age five for children born before 26 weeks (24). A UK model estimated that the incremental cost per preterm child up to 18 years was US$ 35, 471, with substantially higher costs for very preterm infants (17). Additionally, a German claims data analysis highlighted that ambulatory treatment costs for early preterm infants remained elevated during first three years of life (25). These findings collectively emphasize that the economic consequences of preterm birth persist well into childhood and beyond.

In Canada, efforts to reduce neonatal and childhood morbidity include the National Quality Improvement Project of Evidence-Based Practice for Improving Quality (EPIQ-3), which has shown sustainable improvements in outcomes of preterm neonates (26). Recognizing the long-term complications of PTB is essential for ensuring clinical follow-up that enables active prevention, monitoring, and early treatment of health sequelae. This narrative review aims to discuss the common long-term sequelae of PTB affecting various body systems (Table 1), by evaluating existing knowledge and evidence in scientific literature, with a particular focus on their progression and impact during early childhood, adolescence, and adulthood.

**Table 1:** Examples of long-term adverse health impacts of prematurity.

Systems involved	Increased risks
Neurodevelopment	Motor delay
	Cerebral palsy
	Epilepsy
	Developmental delay and intellectual disability
	Autism spectrum disorder
	Attention deficit and hyperactivity disorder
	Neuropsychiatric disorders
	Hearing and vision impairments
Respiratory	Asthma
	Sleep-disordered breathing
	Pulmonary hypertension
Allergy	Allergic rhinitis
	Food allergies
Cardiovascular	Hypertension
	Ischemic heart diseases
	Heart failure
Renal	Acute Kidney injury
	Chronic kidney diseases
	Hypertension
	Hyperfiltration (Supra normal GFR)
	Reduced GFR
	Microalbuminuria
Gastrointestinal	Hernia
	Irritable bowel syndrome (likely)
	Esophagitis (likely)
Endocrine	Growth
	Hypothyroidism
	Diabetes mellitus
Oncological	Hepatoblastoma
	Acute myeloid leukemia
	Sarcoma
	Retinoblastoma
	Germ cell tumors

## 2 Long-term consequences of prematurity

### 2.1 Neurodevelopmental outcomes

The correlation between reduced gestational age and the risk of developing neurodevelopmental disabilities, such as learning problems, poor motor skills, cognitive, hearing, and visual impairments during childhood, is well-documented, particularly among children born extremely preterm (27–31). This association is not surprising, considering that cerebral corticogenesis, the process responsible for the formation of the cerebral cortex, is most active during the third trimester of pregnancy (32, 33). The more preterm, the higher the chance of brain injury and altered trajectory of brain development (i.e., dysmaturation) (34). In contemporary cohorts, despite lower prevalence of severe brain injury such as cystic periventricular leukomalacia, more commonly seen diffuse white matter injury and encephalopathy of prematurity result in abnormal brain maturation (35). For preterm infants, oligodendrocyte precursors which are the predominant cell type in the pre-myelinated white matter, are particularly vulnerable to oxidative stress, inflammation (such as from infection) and hypoxic-ischemic insults (34). The vulnerable developing brain is susceptible to injury during specific windows of development, especially during 23–32 weeks gestation when oligodendrocyte precursors are the most abundant (34). White matter injury of prematurity is also a significant contributor as arrested and altered development of oligodendrocytes result in reduced and altered connectivity of the developing brain (34). Consequently, children born very preterm often exhibit decreased brain volume, surface area, and cortical thickness (32). Additionally, there can be a reduction in neuronal cells due to central nervous system infections or hypoxemic-ischemic insults, both of which can trigger pro-inflammatory cytokines, e.g., interleukin (IL)-6, IL-8, and IL-1β, which are also associated with spontaneous PTB (36–40). Elevated levels of IL-6 may be linked to abnormal brain development and autism, while IL-1β may contribute to neuronal damage (39). Thus, preterm infants confront an increased susceptibility to a range of disorders spanning from motor and developmental to cognitive, visual and hearing loss, epileptic, behavioral, sensory, and neuropsychiatric conditions (31, 41–44). The direct consequences of preterm birth on neurodevelopment necessitate robust clinical monitoring and early interventions to minimize potential risks and optimize long-term health outcomes. Although this section highlights many conditions for which preterm infants have a high risk of developing, it is important to keep in mind that identification of these conditions can often improve functional outcomes through targeted supports and early interventions.

#### 2.1.1 Motor outcomes

Challenges in motor development are common and well known in populations of children born preterm, particularly those born extremely preterm, ranging from delays in sitting or walking to developmental coordination disorder and more significant neuromotor impairment, such as cerebral palsy (CP) (27, 45). CP is a nonprogressive disorder of motor development caused by disturbances in the developing brain. Brain injury in premature infants such as intraventricular hemorrhage (IVH), periventricular hemorrhagic infarction and periventricular leukomalacia increase the risk of motor impairment (46). Most preterm born children who have CP are ambulatory with some assistance or equipment such as orthotics with those more severely affected being non-ambulatory and requiring mobility devices (47). Motor impairments such as developmental coordination disorder are associated with impacts beyond motor aspects of cognitive and behavioral functioning, including associations with decreased academic achievement and higher rates of anxiety (48).

The prevalence of CP is strongly correlated with the severity of prematurity, with approximately 10%–20% of individuals born extremely preterm being affected (27, 45, 49). A population-based study in France examining preterm infants at 2 years of age found that the most common presentation of CP is bilateral CP with spastic diplegia, and the prevalence of CP was 12.4% in those born at GA 24–26 weeks and 2.4% in those born at GA 32–34 weeks (50). Additionally, findings from a systematic review and meta-analysis (12 studies) demonstrated a significant positive association between preterm birth and CP [pooled Odds ratio (OR): 2.9; 95% CI: 2.1; 3.9] (51).

#### 2.1.2 Cognitive outcomes

Children born extremely preterm have increased risk of challenges in cognitive abilities, which include but are not limited to issues with intellectual abilities, working memory, visuomotor integration, self-regulation and executive functions (52). As a result of challenges in these areas, they can have increased difficulties with academic achievement in several areas, including mathematics, reading, and spelling, which may persist throughout their schooling (42, 53). On average, they score 11–13 points lower on IQ scales compared to term-born children (42, 53). Neurodevelopmental outcomes in preterm infants depend on a variety of prenatal, perinatal, neonatal, postnatal and environmental factors, such as their general health, with poorer outcomes observed in very low birth weight (VLBW) infants (54, 55). A longitudinal cohort study in the Netherlands followed the neurodevelopmental trajectories of 705 infants born very preterm (GA <32 weeks) for 19 years (55). The study analyzed separate cohorts of very preterm (VP) infants with VLBW, VP infants without VLBW, and VLBW infants without VP for outcomes such as IQ score, neuromotor function, hearing, behavioral and emotional functioning, and educational achievement. Among the three groups, VP infants without VLBW had the best outcomes, while VLBW infants without VP had the worst outcomes (55). A meta-analysis of 14 studies also found that VP infants with neonatal sepsis were at a higher risk of neurodevelopmental impairment compared to VP infants without sepsis (54).

#### 2.1.3 Communication, social-emotional and behavior conditions: autism spectrum disorder, attention deficit and hyperactivity disorder, and other neuropsychiatric disorders

Language delay is one of the most common challenges that preterm infants face (56). In addition to clinical risk factors, it important to highlight those sociodemographic and structural factors such as racism, lower socioeconomic status, lower caregiver education are associated with negative impact on outcomes on language development (56).

Autism spectrum disorder (ASD), a neurodevelopmental disorder, is likely caused by multiple prenatal, perinatal, and postnatal factors (57, 58). A national Swedish cohort study of over 4 million infants born between 1973 and 2013 found that the prevalence of ASD in all preterm births was 2.1%, with the highest prevalence (6.1%) observed in those born extremely preterm (59).

Another common childhood neurodevelopmental problem is attention deficit and hyperactivity disorder (ADHD). Studies have shown that PTB is a risk factor for ADHD, and the risk increases with each declining week of gestation (60, 61). These findings emphasize the importance of early evaluation, timely detection, and treatment, as well as long-term follow-up for ASD and ADHD, particularly in individuals born extremely preterm.

PTB has been associated with an increased susceptibility to psychiatric conditions, including affective disorders (such as depression and bipolar disorder) and psychosis, in young adults born prematurely (62, 63). Among 1.3 million young patients (>16 years old) recruited in Sweden, those born premature at GA <32 weeks have a higher likelihood of developing psychotic disorders than those born at GA >32 weeks GA (hazard ratio of 2.5 vs. 1.6) (63). These findings align with a Swedish cohort study, which demonstrated a threefold increase in the likelihood of antipsychotic medication prescriptions among 635,933 adults (aged 25.5–34) born extremely preterm (23–27 weeks GA) (64).

#### 2.1.4 Sensory impairments (hearing, vision, and sensory processing)

Children born prematurely have a high risk of hearing impairment, ranging from mild hearing loss to hearing loss requiring cochlear implants (65, 66). Risk factors for hearing impairment include exposure to mechanical ventilation, commonly used ototoxic medications such as furosemide, or aminoglycosides (e.g., gentamicin) (67, 68). Common vision problems in preterm infants include myopia, reduced visual acuity, refractive error, strabismus, abnormal stereopsis, and retinal detachment (65, 69). Although PTB infants are also at increased risk of developing retinopathy of prematurity, problems with vision can still occur in the absence of history of retinopathy of prematurity (70). Additionally, sensory processing challenges, including hypo- and hypersensitivities, are frequently observed in preterm children (71, 72). While trends in both hearing and visual impairments have improved over time due to advancements in neonatal care, screening for these sensory difficulties, followed by interventions such as appropriate feeding strategies and occupational therapies, can significantly improve the child's functional outcomes.

#### 2.1.5 Epilepsy

PTB has been associated with an increased risk of epilepsy in children and young adults, regardless of neurodevelopmental comorbidities (73). A Finland cohort of over 1 million infants, born between 1991 and 2008, found the incidence of epilepsy decreases with increased weeks of gestation (2.5% in the very preterm, 1.1% in the moderate preterm, 0.7% in the late preterm and 0.5% in the term group) (74). The study also documented that the risk of epilepsy was 1.5–4.5 times higher in PTB compared to term infants, followed until seven years after birth or up to 2009 (74). The increased risk of epilepsy due to PTB is long-term, as a Swedish cohort study of over 600,000 adults (age 25–37) found a five-fold increase in the risk of hospitalization due to epilepsy in individuals born extremely preterm compared to those born full-term (75).

### 2.2 Respiratory conditions

Preterm newborns often require respiratory support due to their immature lung development, which can lead to subsequent reduced lung function in early childhood and adulthood (76). Adolescents born preterm have been found to exhibit both reduced lung function and modifications in lung structure (77, 78). A study in Australia, involving over 200 children aged 9–11 years, reported multiple alterations in lung structure and function among children born preterm, including pulmonary obstruction, hyperinflation, diminished forced expiratory volume in one second (FEV_1_) Z-scores, and structural lung changes on CT scans in 92% of preterm children (79). Evidence of airway obstruction was also demonstrated in a Norwegian study of approximately 130 youth aged 10–18 years born extremely preterm, particularly in those who had experienced neonatal bronchopulmonary dysplasia (BPD) (78). Infants with a history of BPD exhibit severely reduced expiratory airflow, which can persist into adulthood (80).

#### 2.2.1 Asthma

Asthma is a common chronic respiratory disease characterized by reversible airway obstruction (81). The most common asthma phenotype in childhood is atopic or Th2-high asthma (81); however it is unclear whether children born preterm who are diagnosed with asthma have a different underlying pathophysiology. A cross-sectional study in the USA, involving 90,721 children aged between 0 and 17 years, revealed that preterm birth was significantly associated with a 1.6 (95% CI: 1.4–1.8) times higher likelihood of developing asthma (15). A study in Norway with 46 subjects reported a higher prevalence of asthma and use of asthma inhalers among adults born extremely preterm (35.6% and 17.4%) (compared to those born at term (6.7% and 2.2%) (82). A large Swedish national cohort study examining asthma medication records of 622,616 patients aged 25–35 years found that young adults born extremely preterm had more than a two-fold increase in the risk of being prescribed asthma medications compared to those born at term (83). A meta-analysis of 147,252 European children up to 10 years old found a significant association between PTB and school-age asthma (pooled OR: 1.4; 95% CI: 1.2, 1.8) (84). Of note, a retrospective cohort study in the USA, examining 6,193 children aged 2–83 months born late preterm, did not find a significant association between late preterm birth and asthma (85).

#### 2.2.2 Sleep-disordered breathing

Obstructive sleep-disordered breathing (SDB) is a condition characterized by intermittent upper airway obstruction during sleep, ranging from primary snoring to obstructive sleep apnea (OSA), with untreated severe OSA potentially leading to right heart failure (86). During early childhood, preterm infants, especially those born before 32 weeks of gestation, are at an increased risk of sleep apnea compared to term-born children (87). A large Swedish cohort study involving over 4 million infants found an inverse association between gestational age at birth and the risk of SDB from childhood to mid-adulthood, indicating that PTB is associated with a greater than 40% increased risk of developing SDB (88). Extremely preterm individuals had a more than two-fold increased risk of SDB in adulthood (88).

#### 2.2.3 Pulmonary hypertension

Bronchopulmonary dysplasia (BPD) is a chronic lung disease that frequently affects preterm infants, particularly those born at <32 weeks of gestation (89). A major complication associated with moderate to severe BPD is pulmonary hypertension (PH) (90), a condition defined by a mean pulmonary arterial pressure > 20 mm Hg, applicable to infants older than 3 months (91). Infants with BPD are at an increased risk of developing PH, with approximately 25% of those with moderate to severe BPD progressing to PH (92). PH in preterm infants with BPD can have profound clinical consequences, including altered ventricular function and heart failure (93). Moreover, PH is associated with adverse outcomes, including impaired somatic growth and neurodevelopmental delays (94, 95). These complications highlight the critical need for early detection, careful monitoring, and proactive management of PH in this vulnerable population (88).

### 2.3 Allergic conditions

PTB has an impact on immune system maturation and can influence the development of immune-mediated diseases, including allergy-related conditions (96, 97). Allergy-related diseases, such as allergic rhinitis and food allergies, are often preceded by immunoglobulin E (IgE) sensitization (98). A longitudinal population-based cohort study that followed 3,522 children prospectively up to 24 years of age found an inverse association between PTB and overall sensitization to common food and inhalant allergens (99). Similarly, a large Swedish national cohort study involving over 600,000 infants, followed up until the ages of 25–37 years to assess the prescription of nasal corticosteroids and oral antihistamines for treating allergic rhinitis, also found a negative association between preterm birth and the risks of developing allergic rhinitis (100). One possible explanation for these findings is that preterm infants may be exposed to allergens earlier, leading to a shift from a TH2 to a TH1 lymphocyte response, which promotes allergen tolerance. However, there is no clear evidence to demonstrate an association of adverse food reactions and PTB. For example, recent studies from Sweden (*n* = 3,522), Italy (*n* = 165), and Japan (*n* = 1,197) identified an inverse relationship between PTB and allergies (99, 101, 102). In contrast, a larger Swedish study involving over one million children found no association between moderate preterm birth and food allergies but reported a negative association between very preterm birth and food allergies (103). Similarly, a Canadian retrospective cohort study involving 13,980 children confirmed the absence of any association between gestational age and food allergies (104). These null findings are supported by a case control study of 160 infants admitted to emergency clinical hospital for children in Romania and a retrospective analysis of 2,116 neonates conducted in Japan (105, 106). In contrast, studies conducted in the USA (*n* = 51,748), China (*n* = 3,437), and Portugal (*n* = 1,217) found a strong positive relationship between PTB and food allergies (107–109). The current evidence does not establish a definitive association between PTB and allergic conditions, particularly food allergies.

Existing studies present conflicting findings, with some suggesting an increased risk of food allergies among preterm infants, while others report a decreased risk. This inconsistency likely stems from limitations in sample sizes within the available literature, which are insufficient to draw robust conclusions. Therefore, future research with significantly larger cohorts of preterm infants is essential to clarify the relationship between PTB and allergic outcomes and to provide a more comprehensive understanding of this potential association.

### 2.4 Cardiovascular conditions

Cardiovascular function is often compromised in PTB due to various factors, including excessive metabolic demands resulting from infection or sepsis, and specific physiological changes (110, 111). Preterm infants commonly exhibit diastolic dysfunction, characterized by impaired relaxation of the heart during the filling phase, even when their systolic left ventricular systolic function is normal. There is an increased dependence on atrial contraction and a compensatory increase in the heart rate in preterm infants. These findings are likely contributors to the known predisposition to cardiovascular disease later in life (112–114).

A study conducted on a cohort of 72 adolescents with a mean age of 13.3 years found that individuals born preterm had statistically significant smaller ventricular size and decreased cardiac mass compared to those born at term (115). Additionally, rapid childhood weight gain observed in preterm infants born with a low ponderal index (body weight-to-height ratio) can place an additional burden on the cardiovascular system and increase the risk of cardiovascular diseases (116). A study in Canada involving nearly 2,000 children with a mean age of 5.6 years found an association between preterm birth and an increase in the composite cardiometabolic risk score (taking into account factors such as waist circumference, triglyceride level, glucose level, systolic blood pressure, and high-density lipoprotein cholesterol level) (116). Lipid disorders (such as elevated cholesterol or triglycerides) have been found to be associated with PTB in a large Swedish national cohort study of more than 2 million people, with a 14% reduction in the risk of lipid disorders for each increment of five weeks of gestation (117).

#### 2.4.1 Hypertension

Hypertension is one of the cardiovascular conditions commonly associated with preterm birth. A large Swedish national cohort study that followed over 600,000 infants into adulthood found that young adults born extremely preterm had a 2.5-fold increased risk of using antihypertensive medications per year compared to those born at term (118). A meta-analysis of 10 studies involving 1,342 infants born preterm (GA 28.8–34.1 weeks) or VLBW (mean birth weight 1,280 g), with their systolic blood pressure measured at age 6.3–22.4 years old, found that patients born preterm or VLBW had higher systolic blood pressure (SBP) by 2.5 mm Hg (95% CI: 1.7, 3.3 mm Hg) than those born term (119). Another meta-analysis of nine cohorts with blood pressure measurements in adults born preterm (GA <37 weeks) at VLBW (birth weight <1,500 g) found that these populations had SBP and diastolic blood pressure 3.4 mm Hg and 2.1 mm Hg higher than adults born term, respectively (120). One multicenter longitudinal study in Finland, involving over 1,700 individuals, found that those born preterm with small-for-gestational age had SBP 7.2 mm Hg higher (95% CI: 2.3, 12.2 mm Hg) than those born at term at the 31-year follow-up (121). The pathophysiology of hypertension in individuals born preterm is not fully understood but may involve inadequate elastin synthesis and altered endothelial cell function associated with PTB (122). Another study of 600 adults in Finland evaluating heart rate variability found that adults born preterm (GA <36 weeks) had decreased vagal control compared to those born term, postulating that such altered autonomic regulatory control may contribute to impaired vascular function and hypertension in adults born preterm (123). It is crucial to emphasize the need for regular BP monitoring in premature survivors of the neonatal intensive care units (NICUs), given the increased risk of developing hypertension later in life. The American Academy of Pediatrics recommends follow-up for hypertension in high-risk populations, including preterm infants, to detect and manage any early signs of elevated BP (124). While office BP measurements in childhood may appear normal, studies have shown that subtle abnormalities, such as a lack of nighttime dipping or increased BP load, can be detected using 24-hour ambulatory blood pressure monitoring (125). These more sensitive measurements may identify early signs of hypertension before it becomes clinically apparent, enabling timely interventions and better long-term cardiovascular outcomes for premature survivors (125).

#### 2.4.2 Ischemic heart diseases and heart failure

PTB is associated with an increased risk of ischemic heart disease and heart failure in adulthood. A large national cohort study in Sweden, involving more than 2 million individuals born preterm between 1973 and 1994, found an inverse association between gestational age and ischemic heart disease in adulthood (126). The study also showed that preterm male and female infants had a 1.4- and 2.0-times higher risk, respectively, of developing ischemic heart disease compared to full-term infants, particularly in older age groups (126). This increased risk is consistent with the presence of cardiometabolic risk factors associated with preterm birth, such as hypertension, lipid disorders, and diabetes mellitus (117, 118, 127). PTB has also been associated with an increased risk of heart failure (3.6-fold in those born 28–31 weeks GA), extending from childhood to adulthood, in large Swedish national cohort studies involving over 2 million people (128). Similarly, another large Swedish cohort study with more than 4 million people found 4.7-fold increased risk in heart failure in adults born extremely preterm (22–27 weeks GA) (129).

### 2.5 Renal conditions

PTB has a significant impact on kidney development (130). The accelerated phase of nephrogenesis, responsible for 60% of nephron formation, occurs during the third trimester of pregnancy and nephrogenesis stops at 36 weeks of GA (130). Therefore preterm neonates have lower nephron endowment, sometimes resulting in smaller kidneys in terms of length and volume compared to full-term neonates (131–134). Autopsy studies revealed that nephrogenesis can continue for approximately 40 days after PTB (135). However, the nephrons developed during this period often exhibit histological abnormalities, such as poor glomerular capillarization, indicating impaired vascularization and non-functionality (136–138).

The compensatory mechanism for the reduced number of functional nephrons is hyperfiltration, which can cause glomerular enlargement. Single-nephron glomerular hyperfiltration, an early pathophysiological feature of chronic kidney disease (CKD), can result in either absolute hyperfiltration with elevated glomerular filtration rate (GFR) or relative hyperfiltration with a normal or reduced GFR (139). However, long-term consequences of this adaptation include sodium retention, hypertension, nephron loss due to sclerosis, and ultimately, the development of CKD due to high glomerular pressure from hyperfiltration can lead to pathological changes including secondary focal segmental glomerulosclerosis, which can manifest as mild albuminuria and subtle reductions in GFR in childhood (140–142). Other complications associated with PTB, such as hemodynamic instability leading to hypoperfusion, the use of nephrotoxic medications (e.g., gentamicin, aminoglycosides, and non-steroidal anti-inflammatory drugs), and systemic or urinary tract infections resulting in acute kidney injury, can further interfere with proper kidney development (143, 144).

Evidence from a large multicenter, multinational retrospective cohort study (Canada, Australia, USA, and India) found that the incidence of acute kidney injury (AKI) varied by gestational age (145). Specifically, 48% of patients with AKI were born between 22 and 29 weeks of gestation, compared to 18% and 37% of patients born between 29 and 36 and ≥36 weeks, respectively (145). Similarly, findings from Preterm Erythropoietin Neuroprotection Trial (PENUT) found an inverse relationship between AKI and GA (24 weeks: 27.8%; 25 weeks: 21.9%; 26 weeks: 13.6%; and 27 weeks: 9.4%) (146). However, studies on long-term renal outcomes in individuals born preterm are limited because renal problems may not manifest until later in life. A systematic review on the effects of prematurity on long-term renal health found that GFR is more affected when populations with preterm birth are measured at longer-term follow-ups, between 7.5 and 20 years of age (147). Similarly, hypertension was found to be associated with preterm birth when populations were followed into adulthood (147). A large Swedish cohort study involving over 4 million people found that prematurity (<37 weeks) overall had a 1.3-fold increased risk of new-onset hypertension at ages 18–29 years, and the risk increased to 2.4-fold (95% CI: 1.8, 3.3) in the population born at extremely preterm gestation (22–27 weeks GA) (148). Another large Swedish national cohort study by Crump et al. involving over 4 million infants, found that adults born preterm had a two-fold increased risk of CKD from childhood to mid-adulthood compared to those born at term, with the association between CKD and preterm birth being strongest during early childhood (0–9 years) (149). Interestingly, adults born at early term (37–38 weeks of gestation) also had a 1.3-fold increased risk of CKD compared to those born at term (149). Likewise, the FANCY (Follow-up of Acute Kidney Injury in Neonates during Childhood Years) study indicates that by age 5, VLBW patients with AKI had a 4.5-fold increased risk of renal dysfunction (95% CI: 1.2, 17.1) compared to VLBW patients without AKI (150). Evidence also shows that preterm infants may exhibit early signs such as mild albuminuria, reduced renal volume and GFR, and elevated blood pressure in early childhood (151–153). Since early renal disease is often asymptomatic, continuous monitoring of BP, serum creatinine, and albuminuria is critical in these high-risk infants (154). Additionally, persistent high BP and albuminuria are risk factors for the progression of CKD (154, 155), and antihypertensive medications like angiotensin-converting enzyme inhibitors can help slow this progression (156).

### 2.6 Gastrointestinal conditions

PTB has a significant impact on the development of the gastrointestinal (GI) tract, leading to various abnormalities and disorders (157). Feeding problems are commonly observed in preterm infants and often persist into childhood, with a higher incidence compared to term-born infants (158, 159). These feeding difficulties may arise from the extended stay in NICUs, and the therapeutic interventions received, which can delay positive oral experiences and hinder the development of a nurturing relationship with the caregiver (158, 159). These feeding difficulties can have adverse effects on the growth and development of preterm infants (159). In connection with managing PTB in NICUs, multiple factors (e.g., prolonged hospital microbial exposure, antibiotic use, and feeding choices) contribute to dysbiosis which can cause immune imbalance (160, 161), an increased risk of gastrointestinal diseases, and interference with other physiological systems (162).

A population-based cohort study in Israel has recruited over 200,000 patients (up to 18 years of age) born between 1991 and 2014 to analyze the digestive morbidity associated with prematurity (163). Digestive morbidities under study included esophageal diseases, diseases of appendix, hernia (inguinal, umbilical, and abdominal wall), inflammatory bowel diseases (IBD), irritable bowel syndrome (IBS) and celiac diseases (163). The study findings revealed a notably higher number of hospitalizations for digestive morbidities among PTB compared to those born at term, with the cumulative incidence rates increasing as gestational age declined (163). However, the authors did not find a statistically significant difference in the occurrence of different types of digestive morbidities, except for hernia (163). Another study (*n* = 2,737) suggested a positive and significant relationship between PTB and IBD (OR of 1.5 for Crohn's disease and 1.3 for ulcerative colitis) (164). Another study that recruited more than 7,000 cases of esophagitis found an association between prematurity and esophagitis in populations diagnosed at 9 years or younger (OR: 6.8; 95% CI: 4.6, 10.0), but the association was not as clear in populations diagnosed at 20 years of age (165). Similarly, a nested case control study in the USA found a positive association between PTB and eosinophilic esophagitis among study subjects aged 18 years and above; however, the results were not statistically significant (OR: 2.6; 95% CI: 0.5, 13.8) (166). The reasons for the association between PTB and esophagitis in early childhood, but not in adulthood, are not clear and warrant further investigation to elucidate the underlying mechanisms for this relationship. Overall, the long-term effects of preterm birth on the GI system are still being studied, and further research is needed to fully understand the specific gastrointestinal conditions and their association with PTB.

### 2.7 Endocrine conditions

#### 2.7.1 Linear growth velocity

Growth is a complex process being affected by intrinsic factors such as chronic health status, nutrition, hormonal profile, medications, socioeconomic status and prolonged hospitalization (167). Although suboptimal growth is a common concern in children born preterm, the relationship between PTB and growth outcomes is complex, with varied and sometimes conflicting findings in the literature. For instance, Hollanders et al. emphasize the importance of distinguishing between VP infants and VLBW infants, highlighting that these groups follow distinct growth trajectories and achieve different final height outcomes (168). Their study suggests that prematurity and low birth weight independently impact growth patterns, emphasizing the need for stratified analyses to elucidate these effects (168). In contrast, Ferguson et al. focus on preterm infants born appropriate-for- gestational-age (AGA), revealing that while growth in this subset may initially lag, catch-up growth is typically observed over time (169). These findings underscore the protective role of AGA birth weight against some of the adverse growth effects of prematurity. These insights align with additional findings from a longitudinal study in New Zealand, which demonstrated that preterm AGA individuals (aged 2–20 years) were slower in reaching their genetically determined height potential compared to those born at term (16). Similarly, a large-scale study involving over 3.2 million children identified an increased risk of stunted growth at 12 and 24 months among late preterm AGA infants compared to those born at term (170). However, a United Kingdom (UK) cohort study involving 204 preterm AGA children and 50 term-born controls found no significant differences in adult height, though preterm children exhibited prolonged catch-up growth periods before attaining their final height (169). Together, these studies demonstrate the variability in growth outcomes influenced by the interplay of gestational age, birth weight, and environmental and genetic factors. Recognizing the multifaceted effects of PTB on growth requires careful interpretation of these findings, particularly considering birth weight appropriateness and the timing of growth assessments. Future research should prioritize examining these interactions.

In addition, there is a complex interplay between prematurity-associated factors and the occurrence of obesity in both childhood and adulthood (171). A systematic review of 19 studies, including 169,439 children, revealed that preterm infants were significantly more likely to develop childhood obesity compared to those born full-term (OR = 1.19, 95% CI: 1.13, 1.26) (172). This increased likelihood of obesity in preterm survivors can be attributed to several factors, including fetal nutrient deprivation, which can lead to metabolic alterations that predispose individuals to obesity later in life (173–175). Furthermore, accelerated weight gain during the neonatal period, often as a compensatory response to early growth deficits, is also a contributing factor (176, 177). The findings from the same review suggest that preterm infants who experience rapid weight gain from birth to the first year of life had a 2.69 times greater risk of developing obesity by ages 8–11 (172), a finding that is consistent with other research showing that fast weight gain in infancy is linked to a 2–4 fold higher risk of obesity (178–181). However, breast milk feeding has been shown to offer a protective effect, reducing the incidence of obesity in preterm infants (182). The nutrients in breast milk support healthy growth and regulate metabolism, potentially mitigating the risk of excessive weight gain in these vulnerable infants (183). These findings underscore the need for careful monitoring of growth patterns in preterm infants and the promotion of breast milk feeding to reduce the long-term risks of obesity.

#### 2.7.2 Thyroid hormone

Thyroid hormones play a crucial role in growth and development during the fetal, neonatal and early childhood period (184, 185). In term births, thyroid hormone levels show distinct elevations and peaks, which are not observed in preterm births (186). Preterm infants, particularly those born very preterm (<32 weeks gestation), are at increased risk of transient hypothyroxinemia, characterized by low serum thyroxine (T4) levels without an elevation in thyroid-stimulating hormone (TSH) (187). This condition arises from the immaturity of the hypothalamic-pituitary-thyroid axis, reduced thyroidal iodine stores, and decreased conversion of T4 to the active triiodothyronine (T3) due to underdeveloped deiodinase activity, resulting in decreased T3 and T4 levels (187). Biochemically, serum T3, total T4 and free T4 are reduced in proportion to the degree of prematurity but without marked elevation of TSH levels; this phenomenon is known as hypothyroxinemia of prematurity (186–188). Reduced T3 and T4 levels in preterm newborns with less than 28 weeks of gestation usually take two to three weeks to reach the peak levels seen in term infants (186–188). This biochemical property differentiates it from congenital hypothyroidism, in which reduced T3 and T4 levels are accompanied by persistently elevated TSH. Distinguishing between the two conditions can be done by measuring persistently low T4 levels with marked elevation of TSH in repeated thyroid function tests at 4 weeks and 8 weeks after birth for early and extremely preterm infants (186–188).

The suppressed thyroid hormone levels in preterm infants could be attributed to several factors, including but not limited to immature hypothalamic-pituitary-thyroid regulation, loss of maternal thyroid hormone transfer in PTB, disease states in which cytokine elevation inhibiting the thyroid function, dopamine use for inotropic support, repeated iodine-based contrast computer-tomography use in infants with congenital heart diseases, and inadequate iodine intake postnatally (188, 189).

Thyroid dysfunction in preterm infants, characterized by suppressed thyroid function, is often present during the neonatal period but may also persist into adulthood. For instance, Delange et al. observed a transient thyroid function deficit in preterm Belgian infants, influenced by prematurity and relative iodine deficiency, which generally resolved by 5–7 weeks, though some developed transient hypothyroidism (190). Similarly, Kim et al. observed that nearly one-fifth of preterm infants born before 32 weeks gestation required levothyroxine treatment, with maternal pregnancy-induced hypertension as a significant risk factor. Notably, almost half of those treated had normal initial thyroid function tests, emphasizing the importance of serial monitoring to identify late-onset dysfunction (191). Furthermore, a large Swedish cohort study involving over 600,000 individuals revealed a 1.60-fold increased risk of medically treated hypothyroidism in young adults born extremely preterm (23–31 weeks gestation), suggesting that the impact of PTB on thyroid function may have long-term implications. These findings emphasize the importance of early screening and monitoring of thyroid function across the lifespan of individuals born preterm, while future research should explore the underlying mechanisms of persistent thyroid dysfunction (192).

#### 2.7.3 Diabetes mellitus

Pancreatic beta cells, primarily formed during the third trimester of pregnancy, may have reduced numbers and functionality in PTB, potentially leading to long-term metabolic consequences (193). An inverse relationship between gestational age and plasma insulin levels has been observed at birth and in early childhood (194). Supporting this, a large Swedish national cohort study involving over 4 million individuals demonstrated that those born preterm had an increased risk of diabetes mellitus (DM) from childhood (1.2-fold increased risk for both type 1 and type 2 DM) to mid-adulthood (1.2-fold increase in type 1 DM and 1.5-fold increase in type 2 DM) compared to those born term (127). The association between PTB and type 2 DM is particularly stronger in adult women (127).

Preterm birth has been associated with an increased risk of both type 1 and type 2 DM multiple studies, though findings vary by gestational age and diabetes type. A Swedish cohort study of 3.6 million children found a 10%–20% increased risk of type 1 diabetes among those born at 33–36 or 37–38 weeks, but a lower risk in those born at <33 weeks, a finding requiring further investigation (195). Conversely, a UK cohort of 3.8 million children and an Australian cohort of 558,633 children reported 15%–30% and 1.4–1.2-fold increased risks of type 1 DM, respectively, among preterm or early-term births (196, 197). For type 2 diabetes, a Finnish cohort of 12,813 adults found a 1.68-fold risk for those born <35 weeks (198), while Swedish and Scottish studies reported 1.6–2.0-fold risks in adults born very preterm (<33 weeks) (199, 200). A meta-analysis of ∼2.2 million participants reported pooled odds ratios of 1.2 (95% CI 1.1–1.2) for type 1 DM and 1.51 (95% CI 1.3–1.7) for type 2 DM, highlighting the consistent association between preterm birth and long-term diabetes risk (201). These consistent findings emphasize the need for continued research to elucidate mechanisms and preventive strategies.

### 2.8 Oncological conditions

Childhood cancer is a serious and devastating condition that affects children and their families (202, 203). Several factors associated with PTB increase the risk of developing certain types of childhood cancer. During their stay in the NICUs, preterm infants are exposed to an array of toxic insults including mechanical ventilation-induced oxidative stress, radiation exposure from diagnostic radiographs, treatment with nitric oxide for pulmonary hypertension, and treatment with oxygen (204–206). A Finnish population-based study found an elevated risk of childhood cancer in those born preterm, particularly in the early preterm cohort, with increased rates of acute myeloid leukemia (AML), germ cell tumors, and retinoblastoma (207). A systemic review on cancer risk in children and young adults born preterm found a consistent association between PTB and an increased risk of hepatoblastoma across multiple studies (208). Low birth weight has also been associated with hepatoblastoma in most studies (209–211), although the underlying mechanisms are unclear. Two meta-analyses of observational studies on prematurity and acute leukemia in childhood suggested that preterm birth is associated with an increased risk of AML (212, 213). The risks of other childhood cancers related to PTB appear to be study- or region-dependent. Some childhood cancers that have been proposed, but not definitively linked to PTB, include soft tissue sarcomas, retinoblastomas, and other gliomas (211). Further research is needed to better understand the underlying mechanisms and to identify effective screening strategies.

### 2.9 Genetic and epigenetic links between preterm birth and long-term morbidity

Emerging evidence suggests that genetic and epigenetic factors may play a critical role in both the etiology of PTB and the long-term health outcomes of survivors (214, 215). Genome-wide association studies (GWAS) have identified genetic variants associated with PTB (215, 216), while epigenetic studies have demonstrated that PTB can lead to lasting changes in DNA methylation and other regulatory mechanisms (217, 218). These genetic and epigenetic alterations may contribute to the risk of PTB and potentially play an independent role in the development of long-term multisystemic diseases (219, 220). Additionally, intergenerational studies indicate that mothers born preterm are more likely to deliver preterm infants, suggesting a heritable component that may also shape long-term health trajectories (221–224). However, whether these same genetic and epigenetic factors directly predispose preterm survivors to multisystemic diseases—such as cardiovascular, metabolic, and neurodevelopmental disorders—remains an area of further exploration. Future GWAS and epigenetic studies are needed to elucidate these complex interactions, which may provide insights into targeted interventions aimed at improving long-term health outcomes in this vulnerable population.

## 3 Discussion

This narrative review comprehensively examines the extensive long-term complications associated with PTB, revealing significant findings across multiple health domains. Our analysis highlights the critical need for continuous follow-up and targeted interventions for individuals born preterm, given their increased susceptibility to a range of health challenges including neurodevelopmental, respiratory, allergic, cardiovascular, renal, gastrointestinal, and endocrine system disorders. The novelty of our findings lies in the comprehensive scope of complications covered, offering a holistic understanding of the long-term health outcomes of PTB.

It is crucial to proactively prevent, monitor, and treat long-term consequences of PTB. By implementing specific practices for preterm infants, such as delivering them in perinatal centers equipped to handle high-risk cases, better short-term outcomes can be achieved compared to transferring them after birth (225). Regular clinical follow-up in pediatric clinics plays a vital role in evaluating and managing the various conditions related to PTB. These evaluations should encompass neurodevelopmental, respiratory, allergic, cardiovascular, renal, gastrointestinal, endocrine, and potential oncological conditions, with particular attention given to extremely preterm infants who are at higher risk. Such comprehensive monitoring allows for early detection and intervention, optimizing the long-term health and well-being of preterm individuals. The holistic approach should extend into the transition from pediatric to adult care, ensuring continuity of care and sustained efforts to manage and minimize the long-term consequences of PTB.

Current long-term follow-up programs for preterm infants often prioritize neurodevelopmental outcomes, but a more holistic, multidisciplinary approach is essential to better support these children and their families. Parents have expressed that their concerns extend beyond neurodevelopmental impairments, highlighting the importance of addressing a broader range of health and developmental factors. A study on parental perspectives regarding the outcomes of very preterm infants found that parents value a balanced approach, encompassing not only neurodevelopment but also other physical and psychological aspects of their child's health (226). This insight underscores the need to shift toward a more inclusive surveillance model that captures a wider spectrum of outcomes, ultimately benefiting the long-term well-being of preterm survivors. Further research is needed to deepen our understanding of pathophysiology underlying the long-term consequences of PTB. By gaining more insights into the mechanisms involved, researchers can develop more effective preventive strategies and targeted treatments to mitigate the impact of PTB on health outcomes.

In summary, our findings advocate for the implementation of targeted interventions and continuous follow-up programs tailored to address the specific health needs of this vulnerable population. A proactive approach that emphasizes prevention, monitoring, and treatment is essential for addressing the long-term consequences of PTB. By implementing specific practices, conducting regular clinical follow-ups, promoting holistic care, and advancing research, it is possible to mitigate the impact of PTB and improve the overall health and quality of life of individuals born preterm.
